# Processing and Compatibility of *Corydalis yanhusuo*: Phytochemistry, Pharmacology, Pharmacokinetics, and Safety

**DOI:** 10.1155/2021/1271953

**Published:** 2021-12-30

**Authors:** Lingyan Wu, Yang Yang, Zhujun Mao, Jianjun Wu, Dan Ren, Bo Zhu, Luping Qin

**Affiliations:** ^1^College of Pharmacy, Shaanxi University of Chinese Medicine, Xianyang 712046, China; ^2^School of Pharmaceutical Sciences, Zhejiang Chinese Medical University, Hangzhou 310053, China

## Abstract

Processed and polyherbal formulations (compatibility) are widely used in traditional Chinese medicine (TCM). However, processing and compatibility may alter the efficacy and safety of herbal medicines, and therefore, evaluating the herbal medicines changes after processing and compatibility is important for their safety. Since *Corydalis yanhusuo* (Y.H.Chou & Chun C.Hsu) W.T.Wang ex Z.Y.Su & C.Y.Wu (Family: Papaveraceae and Genera: *Corydalis*), a traditional medicinal plant in China, Japan, Korea, and other Asian countries, has been used for treating a wide range of medical conditions, it is an ideal representative of studying the effects of processing and compatibility on efficacy and toxicity. In this paper, information was obtained by searching electronic databases, classic books, PhD and MSc dissertations, local conference papers, and unpublished materials prior to July 2021. We provide a summary of the phytochemistry, pharmacology, pharmacokinetics, quality control, and safety of *C. yanhusuo* under various processing or compatibility conditions. Based on our findings, vinegar processing is probably the best *C. yanhusuo* processing method, which could increase the absorption rate of tetrahydropalmatine (THP) in the heart, liver, spleen, lung, and brain tissues and alleviate mice muscle tremors and liver damage caused by *C. yanhusuo*. These results indicate that processing and compatibility can reduce toxicity and increase the efficacy of *C. yanhusuo*. The information provides an expanded understanding of the efficacy and toxicity mechanisms of TCM compounds, which is valuable for industrial production quality control and future drug research.

## 1. Introduction

Prior to incorporation as pharmaceutical ingredients and use as medicinal decoctions in clinics, medicinal plants are processed through a series of traditional Chinese techniques such as washing, cutting, and frying with various excipients [1]. Processing, an essential step for herbal medicinal preparations, is a pharmaceutical process applied for different therapeutic purposes, which can increase efficacy and also reduce toxicity of herbal medicines [2]. Meanwhile, compatibility (polyherbal formulations), which entails combining multiple formulae of traditional Chinese medicine (TCM) (two or more medicines) selectively according to the characteristics of the individual medical plants, is a common practice in TCM aimed at achieving balanced or synergistic effects [3]. Therefore, evaluating the herbal medicines changes after processing and compatibility is important for their safety.

**Figure 1:**
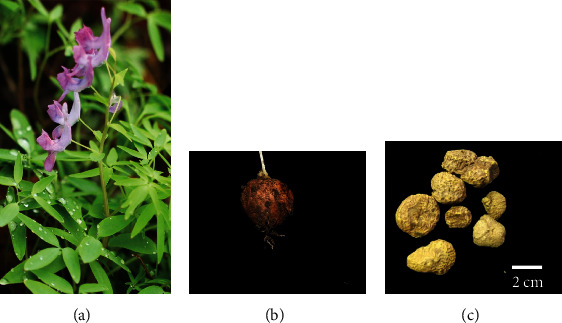
(a) Above ground part of *C. yanhusuo*, (b) tuber of *C. yanhusuo*, and (c) commercial tubers of *C. yanhusuo*.

*Corydalis yanhusuo* (Y.H.Chou & Chun C.Hsu) W.T.Wang ex Z.Y.Su & C.Y.Wu (Synonym: Corydalis turtschaninovii f. yanhusuo Y.H.Chou & Chun C.Hsu; https://www.theplantlist.org), a perennial herb with a 300-year cultivation history, is a typical medicinal raw material that goes through processing and compatibility to achieve a better therapeutic effect (see Figure 1). Its rhizome, known as “Yuanhu,” is often used to treat miscellaneous medical disorders such as pain, insomnia, cardiovascular diseases, hypertension, gastric ulcers, cancer, and inflammation [4]. Additionally, *Corydalis yanhusuo* W.T.Wang (*C. yanhusuo*) is often combined with other medicinal plants such as *Angelica dahurica* in classical TCM prescriptions for enhanced analgesic activity. However, when used in excess (more than 3 g/day), minor toxicity including liver toxicity and muscle tremor in rats have been reported [5]. According to the Chinese Pharmacopoeia 2020 edition, the “standard” processing method is stir-frying *C. yanhusuo* with vinegar. In this process, *C. yanhusuo* and vinegar are combined at a ratio of 5 : 1 and heated at a low temperature until the vinegar has seeped into the medicinal material [6]. Other methods include “stir-frying” with salt, rice wine, clam powder, and glutinous rice, which have been reported in the ancient medical books and local practices, including “Leigong Paozhi Lun” (《雷公炮制论》, Northern-Southern Dynasties, A.D. 588) and “Compendium of Materia Medica” (《本草纲目》, Ming Dynasty, A.D. 1578) [7]. The main bioactive ingredients of *C. yanhusuo* are alkaloids, including tetrahydropalmatine, tetrahydrocoptisine, corydaline, tetrahydroberberine, palmatine, coptisine, and dehydrocorydaline. However, possible interactions and the mechanisms of action of the bioactive compounds present in *C. yanhusuo* are so far unclear. Typical formulation compositions and therapeutic effects of the *C. yanhusuo* are summarized in Supplementary Table S1 (https://www.yaozh.com, last accessed on July 1, 2021).

*C. yanhusuo* processed products and compatible preparations are currently being used in clinical settings; therefore, *C. yanhusuo* is an ideal representative of studying the effects of processing and compatibility on efficacy and toxicity. Changes in pharmacokinetics, which can also explain the changes in drug efficacy, serve as a knowledge base for *C. yanhusuo* processing and compatibility. Processing or compatibility may result in changes in the chemical composition, pharmacological activity, or safety of *C. yanhusuo* and subsequent effects on its application in TCM. In this paper, we provide useful information on the phytochemistry, pharmacological activity, pharmacokinetics, and toxicity of *C. yanhusuo* under various processing and compatibility methods, based on recent studies. This information provides an expanded understanding of the efficacy and toxicity mechanisms of *C. yanhusuo*, which is valuable for industrial production quality control and future drug research, and provides insight into safe and rational use of processed and polyherbal formulations of *C. yanhusuo*.

## 2. Materials and Methods

Information on *C. yanhusuo* was obtained by searching electronic databases, including Web of Science, ScienceDirect, BaiduScholar, PubMed, SciFinder, CNKI, and GoogleScholar, as well as classic books of Chinese herbal medicine, Pharmacopoeia, PhD and MSc dissertations, local conference papers, and unpublished materials prior to July 2021. The keywords were *Corydalis yanhusuo*, processing, compatibility, phytochemistry, pharmacology, pharmacokinetics, safety, and other related words with no time limit (all fields). Due to the lack of data on quality control, the results of several articles were initially considered controversial; subsequently, these articles were discarded because they were scientifically unreliable. In this paper, a total of 41 documents were embraced.

## 3. Phytochemistry

Previous phytochemistry studies have revealed that *C. yanhusuo* has more than 100 compounds, including fatty acids, alkaloids, volatile oils, saccharides, and other compounds [4]. Fatty acids and alkaloids, as dominant compounds in *C. yanhusuo*, showed obvious content change after processing and compatibility.

Fatty acids in *C. yanhusuo* possess anti-inflammatory and antitumor activities [8]. A GC-MS study showed that 7 fatty acids, 2 olefins, and 2 lipids components were identified in raw *C. yanhusuo* and vinegar *C. yanhusuo* at different concentrations. Among the 4 fatty acids with less than 20 carbon atoms (linolelaidic acid methyl ester; methyl oleate; methyl palmitate; methyl stearate) were more abundant in raw *C. yanhusuo*, while 3 fatty acids with more than 20 carbon atoms (methyl arachidate; methyl behenate; methyl lignocerate) were more abundant in vinegar *C. yanhusuo*. Unlike in raw *C. yanhusuo*, ethyl 4, 5, 6, 7-tetrahydro-3-methyl-2-formate indole could not be detected in vinegar processed *C. yanhusuo*. Therefore, further studies are needed to establish whether 4, 5, 6, 7-tetrahydro-3-methyl-2-carboxylic acid ethyl indole can be used as an indicator for the presence of raw *C. yanhusuo* and vinegar *C. yanhusuo* [9].

**Figure 2:**
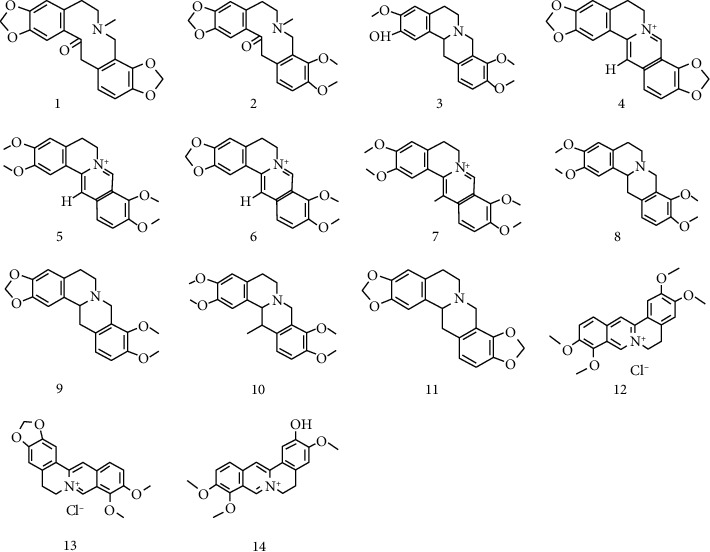
Alkaloids isolated from *C. yanhusuo*.

**Figure 3:**
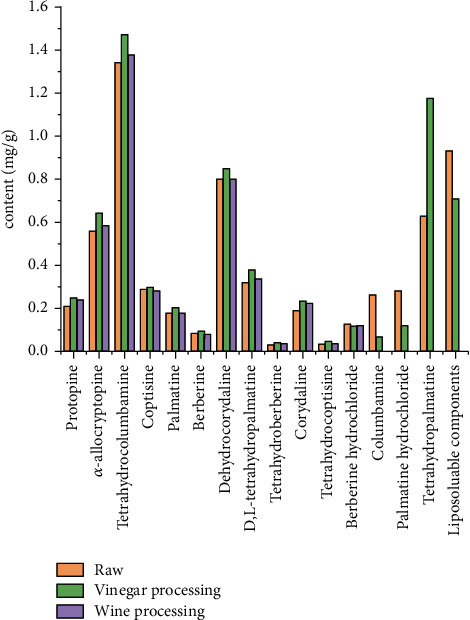
Quantitative changes on the content of *C. yanhusuo* compounds under different processing methods.

Alkaloids are the main compounds of *C. yanhusuo* and have been confirmed to have significant analgesic, anti-inflammatory, and antitumor activities [10, 11]. Their typical structures are shown in Figure 2. The auxiliary vinegar used in processing can be combined with *C. yanhusuo* alkaloids to form salt and increase their solubility, and the dissolution rate of alkaloids is improved; therefore, vinegar processing has been shown to result in increased amounts of protopine and tetrahydrocoptisine [12]. On the other hand, the amounts of berberine hydrochloride, palmatine hydrochloride, and dehydrocorydaline are significantly decreased during vinegar processing; this may be due to the decomposition of quaternary amine alkaloids in *C. yanhusuo* after vinegar boiling [12, 13]. The influence of processing methods on the content of *C. yanhusuo* is compounded as shown in Figure 3 and Table 1. However, related research on some *C. yanhusuo* compounds is still at the initial stages. For instance, while volatile oils and polysaccharides are major active compounds in *C. yanhusuo* and possess anti-inflammatory, antibacterial, antitumor, and cerebral ischemia protective effects, so far, there are no studies on their possible changes before and after processing. Therefore, in future studies, it is necessary to evaluate the content, pharmacology, and safety of these components in processed *C. yanhusuo*. Based on the benefits offered by vinegar processing (e.g., increasing the content of effective ingredients in *C. yanhusuo*) and its long history in TCM, we believe that it is the most suitable method for processing *C. yanhusuo* [13, 14].

## 4. Pharmacological Activities

Owing to its wide range of pharmacological activities, *C. yanhusuo* has been used as a tonic for treating pain [15], insomnia [16], drug addiction [17], and hypertension [18]. In TCM, processed *C. yanhusuo* is believed to have superior pharmacological efficacy compared with raw *C. yanhusuo*. Rat (NIH species, weight 18–22 g, female) was orally administered with vinegar *C. yanhusuo* extracts (10 g/kg) for 6 consecutive hours to test analgesic effect. The results showed that there was significant reduction in the number of writhing reactions (from 10.3 ± 3.9 times/15 mins to 7.8 ± 2.8 times/15 mins) and raise in the pain threshold (from 15.4 ± 4.6 s to 43.7 ± 5.2 s) compared with raw *C. yanhusuo* (10 g/kg) and positive drug indomethacin (1 mg/kg), indicating that vinegar *C. yanhusuo* has better analgesic effects (*P* < 0.05) [19]. However, the reliability of the study is limited by the lack of information on the content changes of *C. yanhusuo* constituents responsible for analgesia or an explanation of the analgesia mechanism. Rat (NIH species, weight 18–22 g, half male and female) was orally administered with vinegar *C. yanhusuo* extracts (4 g/kg) for 3 consecutive days to test anti-inflammatory effect. The results showed that there was significant reduction in ear swelling rate (from 8.8 ± 2.7 to 4.1 ± 1.2), as compared to the raw *C. yanhusuo* (4 g/kg), suggesting that vinegar *C. yanhusuo* has better anti-inflammatory effect [19]. Yuanhu Zhitong prescription (consists of *C. yanhusuo* and *Angelicae dahuricae*; YHZT) was reported to have better analgesic effect compared with *C. yanhusuo* used individually [20].

In addition, polyherbal *C. yanhusuo* with other TCM, such as *Curcuma longa* and *Melia toosendan*, has enhanced the antitumor and antigastric ulcer effects as compared to single *C. yanhusuo*. Rat (SD species, weight 180–220 g, male) was orally administered with Jinlingzi powder extracts (0.13 g/mL) (a combination of *C. yanhusuo* and *Melia toosendan*) for 12 consecutive days to test treatment effects on gastric ulcer. The dose volume was 1 mL/100 g according to the weight of the rats. The results showed that there was significant reduction in N-terminal brain natriuretic peptide precursor (NT) and increased interleukin-8 (IL-8), tumor necrosis factor-*α* (TNF-*α*), platelet-activating factor (PAF), thromboxane B2 (TXB2), and vascular endothelial growth factor (VEGF) in serum, neutral neutrophil activation, which was as effective as the positive drug omeprazole (0.36 mg/mL). The results were statistically significant (*P* < 0.05), indicating that the Jinlingzi powder extracts had a significant effect on the treatment of the ulcer [21]. This is the first time to elucidate the rationality of the prescription in view of pharmacokinetic and pharmacological effects. In addition, YanhusuoSan (a combination of *C. yanhusuo* and *Curcuma longa*) extract-containing medium (100 *μ*L) treatment on MDA-MB-231 cells for 48 h reduced the cell invasion ability, suppressed the level of phospho-extracellular regulated protein kinases (p-ERK), and exhibited the strongest anticancer cell proliferation effect at the ratio of 3 : 2, which provided a plausible molecular basis of antitumor effect of *C. yanhusuo* and *Curcuma longa* [22].

In the future, reasonable quality content standards for nonalkaloids such as volatile oils, organic acids, and polysaccharides are essential, which can help establish a credible theoretical and experimental foundation for *C. yanhusuo* research.

## 5. Pharmacokinetics Studies

### 5.1. Absorption

Changes in efficacy and toxicity of processed and compatible *C. yanhusuo* can also be explicated by the altered pharmacokinetics properties. So far, pharmacokinetics research has mainly focused on *C. yanhusuo* alkaloids. Additionally, beagle dog (12 ± 2 kg, male) was orally administered with YHZT extracts (0.15 g/kg) to detect pharmacokinetics parameters. The results showed that there was significant (*P* < 0.001) raise in the Cmax, *t*_1/2_, AUC_0-∞_, and AUC_0-*t*_ of dehydrocorydaline, tetrahydropalmatine, protopine, palmatine, columbamine, and berberine compared to raw *C. yanhusuo* (0.0486 g/kg), and the *t*_max_ of the two groups was shorter than 1 h. The above results suggest that vinegar processing and compatibility have longer elimination time and better bioavailability [23]. Moreover, the average plasma concentration curves of these compounds showed multipeak phenomenon, which may be related to distribution, reabsorption, and enterohepatic circulation [24]. Additionally, the polyherb of *C. yanhusuo* and *Angelicae dahuricae* is proved to increase the under curve area (AUC_0-*t*_), AUC_0-∞_, and *C*_max_ of CDL and THP, as well as delay mean residence time (MRT_0-*T*_ and MRT_0-∞_) of CDL, suggesting a synergistic effect between alkaloid and coumarin, which is related to their increased absorption [20]. Despite the differences in experimental design, the results of the above studies showed that compounds in *C. yanhusuo* were easily absorbed and slowly eliminated. Only few reports have focused on the effects of pharmacokinetics characteristics on the therapeutic efficacy and toxicity after *C. yanhusuo* processing, and the relationship among the pharmacokinetics, therapeutic effect, and toxicity of processed *C. yanhusuo* remains unclear.

### 5.2. Distribution

Rat (Wistar species, 250 ± 20 g, male) was orally administered with processed *C. yanhusuo* extracts (10 g/kg) for 12 consecutive hours to compare the distribution of THP, dehydrocorydaline (DHC), and protopine in rat tissues. The ethanol extract of the unprocessed *C. yanhusuo* (17.75 g/kg) and THP (6.45 mg/kg), DHC (26.10 mg/kg), and protopine (4.52 mg/kg) pure compound were used as the controls. The results showed that there were various effects on the *T*_max_ and MRT of DHC, THP, and protopine in processed *C. yanhusuo*, and the effect varies in different tissues and among different bioactive compounds indicating that differently processed *C. yanhusuo* products should be selected according to the specific disease condition and the affected organs [25].

However, the above study mainly focused on THP, DHC, and protopine consequently limiting comprehensive utilization and development of *C. yanhusuo* resources. Therefore, studies on the pharmacodynamics and pharmacokinetics of other alkaloids, such as protoberberine, isoquinoline, and nonalkaloids including steroids and organic acids, are required; this will lay a solid foundation for the follow-up research and development of new drugs.

### 5.3. Metabolism

*In vivo* studies showed that differentially processed *C. yanhusuo* formulations were metabolized differently. The elimination of compounds in *C. yanhusuo* in rats is a two-compartment model [26]. Rat (Wistar species, 200–250 g, male) was orally administered with processed *C. yanhusuo* extracts (1.2 g/mL) for 24 consecutive hours to test the metabolism of THP, DHC, and protopine. The ethanol extract of the unprocessed *C. yanhusuo* (1.2 g/kg) and THP (0.516 mg/kg), DHC (0.361 mg/kg), and protopine (1.224 mg/kg) pure compound were used as the controls. The results showed that there was significant raise in the elimination half-life of THP, protopine, and DHC compared with raw *C. yanhusuo*, and mean residence time was more than 6 h, indicating that processing could delay the elimination of THP, protopine, and DHC [27, 28]. However, further studies on the specific mechanism of the synergistic effect are needed.

## 6. Quality Control

Tetrahydropalmatine is one of the alkaloids selected as a phytochemical marker for *C. yanhusuo* preparations quality control in the Chinese Pharmacopoeia. The Chinese Pharmacopoeia suggested that the quality of *C. yanhusuo* preparations should be based on THP properties, microscopic details, and its thin layer chromatography results. When evaluating *C. yanhusuo* quality using the hot dipping method together with the alcohol-soluble determination method, the amount of THP in *C. yanhusuo* should not be less than 0.04% (Chinese Pharmacopeia, 2020) [6]. However, using THP as the only quantitative marker may be insufficient in assessing the quality of *C. yanhusuo*, due to the impacts of other *C. yanhusuo* alkaloids on its physiological activity. Therefore, trahydroberberine and corydalis accounting for the analgesia, sedative, and hypnotic properties should also be considered for quality evaluation.

Different *C. yanhusuo* bioactive components, especially alkaloids, have been identified using highly sensitive technologies such as HPLC before and after processing [29]. For instance, the content of protopine, THP, CDL, berberine hydrochloride, and palmatine hydrochloride in *C. yanhusuo* was assessed using HPLC, and the average recovery was 100.12%∼100.98% (RSD = 1.05%∼1.90%, *n* = 9), suggesting that the HPLC method is an efficient quality control method for *C. yanhusuo* compounds [30]. Q-marker, a newly proposed concept of TCM quality control, recently used IP assays combined with chemical analysis, biosynthesis analysis, drug metabolism, and network pharmacology to identify the Q-markers of *C. yanhusuo*. Interestingly, the Q-markers were found to be highly correlated with multiple *C. yanhusuo* biological activities, confirming the approach efficiency as a quality control method for *C. yanhusuo* before and after processing. However, there is still no suitable method for identifying Q-markers in *C. yanhusuo* complex compatibility system [31].

Dao-di herbs are medicinal herbs produced in specific areas, which have a long traditional use history and excellent medicinal efficacy [32]. An HPLC test on *C. yanhusuo* preparations from 15 production areas showed that the concentrations of CDL and THP were between 0.08–0.44% and 0.03–0.2%, respectively, indicating that the alkaloid content in rhizomes differs significantly between samples from different habitats [33]. According to the “BencaoYuanshi” (《本草原始》) (Ming Dynasty, A.D. 1612), the best-quality medicinal *C. yanhusuo* is known as “Yuanhu” (Dao-di herbs), and it is found in Zhejiang Province. Nonetheless, further studies on the *C. yanhusuo* quality control evaluation are needed. Since no standard quality control methods and indicators are available, further studies incorporating a variety of alkaloids as potential candidates for *C. yanhusuo* quality evaluation index need to be conducted.

## 7. Safety

Based on previous clinical and animal trials, oral administration of raw *C. yanhusuo* extracts (more than 1–3 g/day) or total *C. yanhusuo* alkaloids (473.36 mg/kg) induced liver damage, muscle tremors, and renal hemorrhage in mice, indicating that raw *C. yanhusuo* extracts and total *C. yanhusuo* alkaloids have mild toxicity [34]. The liver damage may be due to the effects of high-dose *C. yanhusuo* including disordered energy metabolism and increased hepatocyte membrane permeability that facilitated the release of intracellular transaminase into the blood, eventually leading to liver cell swelling, acid change, necrosis, and inflammatory cell infiltration [35]. However, the mechanism through which *C. yanhusuo* induces muscle tremor and renal hemorrhage has not been reported, and therefore, further studies are needed.

The hepatotoxicity of total alkaloids in *C. yanhusuo* can be reduced by stir-baking with vinegar combined with *Angelica sinensis* and *Curcumae longa*. A GC-MS study found that hepatotoxicity was significantly decreased with the decreased content of total alkaloids in vinegar processed *C. yanhusuo*; this supported earlier reports that traditional processing could reduce toxicity [36]. Another experiment also confirmed that vinegar processing decreased the solubility of total *C. yanhusuo* alkaloids and reduced toxicity [7, 37]. However, neither of the above studies identified the specific toxic components in *C. yanhusuo*. In addition, oral administration of polyherbal *C. yanhusuo*, *Angelica sinensis*, and *Curcumae longa* significantly alleviated mice muscle tremors and reduced hepatotoxicity [38]. However, the specific mechanism through which the combination reduced toxicity has not been clearly explained. According to the toxicity findings mentioned above, the toxicity of *C. yanhusuo* can be reduced by compatibility and dosage control. Meanwhile, oral administration of *C. yanhusuo* extracts (1–3 g/day) for 1–3 weeks is recommended as the optimal dosage for treatment of acute pain, cancer, and cardiovascular diseases [6]. *C. yanhusuo* is not recommended for patients with “Qi”-associated physical weakness with high body temperature, low levels of body fluids, acute abdominal pain, menorrhagia, and blood deficiency. Clinical studies have also reported that *C. yanhusuo* can increase the toxicity of *Strychnos nux-vomica*, which could be due to the formation of new toxic components as a result of combining *C. yanhusuo* and *Strychnos nux-vomica*. Therefore, *C. yanhusuo* and *Strychnos nux-vomica* should not be used concurrently [39]. In addition, after administration of THP pure compound, several cases were reported that THP possessed poison [40]. In this paper, we found that the concentration of THP in the tissues decreased rapidly after reaching the peak concentration and was only 1/17∼1/49 of *C*_max_ after 12 h, indicating that administration of *C. yanhusuo* instead of THP pure compound will not cause the accumulation of THP in the tissue [25].

In conclusion, raw *C. yanhusuo* extracts induce mild hepatotoxicity, mouse spasms, convulsions, and renal hemorrhage. Orally administered raw *C. yanhusuo* extracts and total alkaloids in *C. yanhusuo* should not exceed 3 g/day and 473.36 mg/kg, respectively. Vinegar *C. yanhusuo* processing and combining *C. yanhusuo* with other medicinal plants are recommended approaches for reducing toxicity. However, *C. yanhusuo* toxic substances have been classified in a large category, namely, total alkaloids, and the specific toxic substances are still not clear, which requires further studies.

## 8. Conclusions and Perspectives

This review summarizes the phytochemistry, pharmacology, pharmacokinetics, quality control, and safety of *C. yanhusuo* under various processing and compatibility methods. Based on our findings, compared with raw *C. yanhusuo*, vinegar processing or polyherbal *C. yanhusuo* with *Angelica dahurica* showed increased analgesic effects, alleviated liver damage and mice muscle tremor caused by *C. yanhusuo*, and promoted the absorption of THP in the liver, suggesting that vinegar processing and combining *C. yanhusuo* with *Angelica dahurica* are the best processing and compatibility methods, respectively.

*C. yanhusuo* has been used for a long time in Asia as an analgesic, antitumor, anti-inflammatory, and cardiovascular protective agent. Several studies on rational processing, design, rational application mechanism of action, and toxicity of *C. yanhusuo* have so far been carried out. However, there are noticeable knowledge gaps that require further studies. First, there is no comprehensive research on synergism and attenuation effects of *C. yanhusuo* preparations; it is not clear whether new components are found after processing or compatibility, and information on the absorption, distribution, metabolism, and mechanism of action of new components is not available. Additionally, the mechanism of interaction between active components in *C. yanhusuo* compatibility preparations has not been clarified. Second, the effects of *C. yanhusuo* processing and compatibility on toxicity evaluation and toxicity mechanism remain unclear. Studies have shown decreased hepatotoxicity induced by *C. yanhusuo* with decreased content of total *C. yanhusuo* alkaloids in vinegar processed *C. yanhusuo*. However, the specific toxic alkaloid compounds and toxic metabolites are still unknown. In addition to the analgesic and sedative effects, *C. yanhusuo* has been reported to cause coronary artery dilation, lower blood pressure, and antiarrhythmia. The effects of processing and compatibility on these pharmacological effects need to be studied. Moreover, most studies are based on animal models, and therefore, clinical studies are needed to verify the therapeutic efficacy of processed *C. yanhusuo*. From the traditional view, processing increased the therapeutic effects of *C. yanhusuo*, while based on the findings, raw *C. yanhusuo* has been proved to have better antitumor activities than processed *C. yanhusuo*; this needs to be further investigated before clinical application. Finally, the vinegar and wine *C. yanhusuo* processing methods are the most commonly used and studied processing methods, which have limited the chances of exploring other processing methods. Due to several influencing factors, it is difficult to achieve unification with the processing technology, and consequent standardization of processing and quality control of TCM are also challenging. Beyond the alkaloids, more active compounds need to be examined during *C. yanhusuo* control quality, and quality standard of processed *C. yanhusuo* and its compatibility need to be established; this will help to ensure the optimal quality, efficacy, and safety of *C. yanhusuo* preparations.

## Figures and Tables

**Table 1:** Quantitative changes on the content of *Corydalis yanhusuo* compounds under different processing methods.

Number	Compounds	Processing method	Processing content (mg/g)	Reference
1	Protopine	None	0.216	[12]
		Stir-baking with vinegar	0.249↑	
		Stir-baking with rice wine	0.241↑	
2	*α*-allocryptopine	None	0.559	[12]
		Stir-baking with vinegar	0.644↑	
		Stir-baking with rice wine	0.585↑	
3	Tetrahydrocolumbamine	None	1.340	[12]
		Stir-baking with vinegar	1.479↑	
		Stir-baking with rice wine	1.379↑	
4	Coptisine	None	0.290	[12]
		Stir-baking with vinegar	0.300↑	
		Stir-baking with rice wine	0.281↓	
5	Palmatine	None	0.180	[12]
		Stir-baking with vinegar	0.177↓	
		Stir-baking with rice wine	0.204↑	
6	Berberine	None	0.084	[12]
		Stir-baking with vinegar	0.078↓	
		Stir-baking with rice wine	0.092↑	
7	Dehydrocorydaline	None	0.800	[12]
		Stir-baking with vinegar	0.797↓	
		Stir-baking with rice wine	0.851↑	
8	D, L-tetrahydropalmatine	None	0.321	[12]
		Stir-baking with vinegar	0.339↑	
		Stir-baking with rice wine	0.381↑	
9	Tetrahydroberberine	None	0.032	[12]
		Stir-baking with vinegar	0.040↑	
		Stir-baking with rice wine	0.036↑	
10	Corydaline	None	0.189	[12]
		Stir-baking with vinegar	0.224↑	
		Stir-baking with rice wine	0.233↑	
11	Tetrahydrocoptisine	None	0.033	[12]
		Stir-baking with vinegar	0.036↑	
		Stir-baking with rice wine	0.044↑	
12	Berberine hydrochloride	None	0.128	[13]
		Stir-baking with vinegar	0.120↓	
		Stir-baking with rice wine	0.118↓	
13	Columbamine	None	0.262	[13]
		Stir-baking with vinegar	0.068↑	
14	Palmatine hydrochloride	None	0.282	[13]
		Stir-baking with vinegar	0.120↓	
		Stir-baking with vinegar	1.177↑	
15	Liposoluble components	None	0.932	[9]
		Stir-baking with vinegar	0.711↓	

PubChemCID: protopine: 4970; *α*-allocryptopine: 98570; tetrahydrocolumbamine: 440229; coptisine: 72322; palmatine: 19009; berberine: 2353; dehydrocorydaline: 34781; tetrahydroberberine: 34458; corydaline: 10130; tetrahydrocoptisine: 6770; berberine hydrochloride: 12456; columbamine: 72310; palmatine hydrochloride: 73442. CAS registry number: D, L-tetrahydropalmatine: 10097-84-4 (↑ denotes that the content of corresponding compounds increased compared with the raw *C. yanhusuo*; ↓ denotes that the content of corresponding compounds decreased compared with the raw *C. yanhusuo*).

## Supplementary Materials

Supplementary Materials Supplementary Table S1 provides examples of traditional Chinese medicine prescriptions containing *Corydalis yanhusuo* W.T.Wang.
