# Supplementary material for: Processing and Compatibility of Corydalis yanhusuo: Phytochemistry, Pharmacology, Pharmacokinetics, and Safety
Source: Evid Based Complement Alternat Med. 2021 Dec 30;2021:1271953. doi: 10.1155/2021/1271953 (PMC8739176; doi:10.1155/2021/1271953)
Supplement: Supplementary Materials — Supplementary Table S1 provides examples of traditional Chinese medicine prescriptions containing Corydalis yanhusuo W.T.Wang. [file 1271953.f1.docx]

Supplementary Table S1: Examples of traditional Chinese medicine prescriptions containing *Corydalis yanhusuo* W.T.Wang

| Preparation name | Composition | Role of *C. yanhusuo* in  prescription | Traditional and clinical uses | Refs. |
| --- | --- | --- | --- | --- |
| Cui Sheng Shen Xiao Qi Sheng San | *Corydalis yanhusuo, Commiphora myrrha., Angelica dahurica, Curcuma longa, Angelica sinensis, Cinnamomum cassia* | Leading role | Treat dystocia | Fu Ren Da Quan Liang Fang 《妇人大全良方》 |
| Zhong Jing Wei Ling Wan | *Corydalis yanhusuo, Cinnamomum cassia, Ostreagigas, Foeniculum vulgare, Amomum villosum, Alpinia officinarum , Paeonia lactiflora, Glycyrrhiza uralensis* | Leading role | Treat weak spleen and stomach, loss of appetite, cold congealing stomachache, abdominal distension | Chinese Pharmacopoeia (2020) |
| Lan Wei Hua Yu Tang | *Corydalis yanhusuo, Floslonicerae, Meliae toosendan, Rheum officinale, Cortex moutan, Semen persicae, Aucklandia lappa* | Leading role | Treat the initial stage of appendicitis of stagnation type. fever, abdominal distension, abdominal pain, right lower abdominal local tenderness, rebound pain | Neoacuteabdomenology 《新急腹症学》 |
| Jin Ling Zi San | *Corydalis yanhusuo ,Meliae toosendan* | Leading role | Treat atrophic gastritis, liver depression and fire syndrome, heart, chest, ribs and abdomen pain | Tai Ping Sheng Hui Fang 《太平圣惠方》 |
| Yuan Hu Zhi Tong Pian | *Corydalis yanhusuo, Angelica dahurica* | Leading role | Treat gastric pain, hypochondriac pain, headache and dysmenorrhea due to ‘Qi’ stagnation and blood stasis | Chinese Pharmacopoeia (2020) |
| Bi Ying Tang | *Corydalis yanhusuo, Cyperus rotundus, Artemisia argyi, Angelica sinensis, Amomum villosum, Zingiber officinale* | Leading role | Treat cold evils in the heart and spleen, ‘Qi’ and blood discord, chest pain | Za Bing Yuan Liu Xi Zhu 《杂病源流犀烛》 |
| Bei Ji Wan | *Corydalis yanhusuo, Aucklandia lappa, Faeces togopteri, Aquilaria sinensis, Croton tiglium* | Leading role | Treat sudden bloating, sudden pain, and urination | Huo Ren Fang  《活人方》 |
| Ban Xuan Wan | *Corydalis yanhusuo ,Mylabris phalerata* | Leading role | Treat pregnancy miscarriage, symptomatic symptoms, and ‘Qi’ and blood pain | Yi Xue Ru Men 《医学入门》 |
| Qing Re Tiao Xue Tang | *Corydalis yanhusuo, Angelica sinensis, Ligusticum chuanxiong, Paeonia lactiflora, Rehmannia glutinosa ,Coptis chinensis, Cyperus rotundus, Semen persicae, Carthamus tinctorius, Cortex moutan, Curcuma aeruginosa* | Leading role | Treat dysmenorrhea, bellyache, chronic pelvic inflammatory disease, adenomyosis of the uterus | Gu Jin Yi Jian 《古今医鉴》 |
| Bu Xue Ding Tong Tang | *Corydalis yanhusuo, Angelica sinensis, Ligusticum chuanxiong, Rehmannia glutinosa, Paeonia lactiflora, Cyperus rotundus, Semen persicae, Carthamus tinctorius, Citrus reticulata, Alisma orientale, Cortex moutan.* | Leading role | Treat postpartum bleeding abdominal pain | Diseases rejuvenation 《万病回春》 |
| Fu Yuan Tong Qi San | *Corydalis yanhusuo, Citrus reticulate, Pharbitis nil, Glycyrrhiza uralensis, Aucklandia lappa, Foeniculum vulgare, Manis pentadactyla, Angelica sinensis, Boswellia carterii, Commiphora myrrha* | Supporting role | Treat sore, carbuncle, chilling pain, pus ulcerated or not ulcerated and hernia, ‘Qi’ stagnation and pain | Yi Xue Ru Men 《医学入门》 |
| Shao Fu Zhu Yi Tang | *Corydalis yanhusuo , Foeniculum vulgare, Zingiber officinale, Commiphora myrrha,, Angelica sinensis, Atractylodes chinensis, Ligusticum chuanxiong, Cinnamomum cassia, Typha angustifolia, Faeces togopteri, Paeonia veitchii* | Supporting  role | Treat less abdominal mass, less abdominal distention, menstrual lumbar acid, lower abdominal distention, metrorrhagia | Yi Lin Gai Cuo 《医林改错》 |
| Ge Xia Zhu YuTang | *Corydalis yanhusuo, Faeces togopteri, Angelica sinensis, Ligusticum chuanxiong, Semen persicae, Cortex moutan, Paeonia veitchii, Lindera aggregata, Glycyrrhiza uralensis, Cyperus rotundus, Carthamus tinctorius, Citrus aurantium* | Supporting role | Treat subphrenicstasis blocking ‘Qi’block, chronic active hepatitis, hematoporphyria, diabetes, ectopic pregnancy and infertility | Yi Lin Gai Cuo 《医林改错》 |
| Yan Hu Suo San | *Corydalis yanhusuo, Angelica sinensis, Typha angustifolia, Paeonia veitchii, Cinnamomum cassia, Boswellia carterii, Commiphora myrrha, Curcuma longa, Aucklandia lappa, Glycyrrhiza uralensis* | Supporting role | Treat ‘Qi’ stagnation and blood stasis, abdominal distension and pain | Ji Sheng Fang 《济生方》 |
| He Yin Tong Qi San | *Corydalis yanhusuo, Angelica sinensis, Salvia miltiorrhiza, Cyperus rotundus, Ligusticum chuanxiong,, Citrus reticulata, Citrus aurantium, Curcuma wenyujin, Pinellia ternata, Aucklandia lappa, Foeniculum vulgare* | Supporting role | Treat internal injury of trunk, blockage of ‘Qi’ and blood, chest, lumbar and abdominal distension, poor breathing | Traditional Chinese Medicine Traumatology《中医伤科学讲义》 |
| Tao Ren Hong HuaJian | *Corydalis yanhusuo, Salvia miltiorrhiza, Paeonia veitchii, Semen persicae, Carthamus tinctorius, Cyperus rotundus,, Citrus reticulata, Angelica sinensis, Ligusticum chuanxiong, Rehmannia glutinosa* | Supporting role | Treat heart blood stasis. palpitation, chest tightness and discomfort | Surin Medical case  《素庵医案》 |
